# Supplementary material for: Cortical neural dynamics unveil the rhythm of natural visual behavior in marmosets
Source: Commun Biol. 2022 Feb 3;5:108. doi: 10.1038/s42003-022-03052-1 (PMC8814246; doi:10.1038/s42003-022-03052-1)
Supplement: Supplementary file 2 — Supplemental Information [file 42003_2022_3052_MOESM2_ESM.pdf]

Supplementary Information

Cortical neural dynamics unveil the rhythm of natural visual behavior in marmosets

Takaaki Kaneko*, Misako Komatsu, Tetsuo Yamamori, Noritaka Ichinohe, Hideyuki Okano*

*Corresponding authors.
Email: kaneko.takaaki.6w@kyoto-u.ac.jp (T.K.), hidokano@a2.keio.jp (H.O.)

This PDF file includes:

Supplementary Figure 1. Statistical assessment of perisaccadic activation/suppression pattern of active vision

Supplementary Figure 2. Perisaccadic modulation time-locked with saccade or fixation onset

Supplementary Figure 3. Trajectory of the high-gamma signal on the cortical surface for individual data

Supplementary Figure 4. Neural dynamics of passive visual perception

Supplementary Figure 5. Anatomical connectivity from an open database

Supplementary Figure 6. Granger causality analysis

Supplementary Figure 7. Signal time course of visual and non-visuomotor areas

Supplementary Figure 8. Temporal dynamics of whole brain activity in relation to saccade interval (only visual-responsive areas)

Supplementary Figure 9. Temporal dynamics of whole brain activity including positive and negative components

Supplementary Figure 10. Individual data for distinctive activity profiles at fixation termination for different fixation durations

Supplementary Table 1. Mapping cortical annotation to brain regions

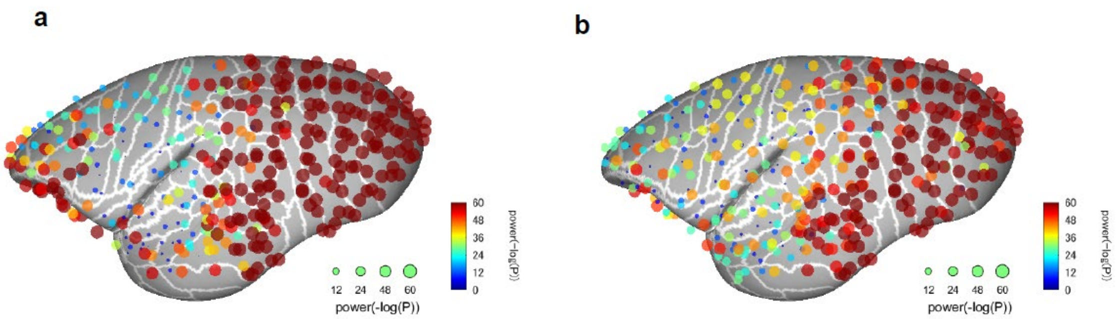

Supplementary Figure 1. Statistical assessment of perisaccadic activation/suppression pattern of active vision

The *p*-values of high-gamma activity magnitude were obtained by a randomization test. Size of the marker indicate the negative log of the *p*-value. Signal modulation (as shown by the *z*-score) was quite low across most of the electrodes, though the *p*-value obtained by the randomization test showed the modulation was statistically robust and reliable. (**a**) *P*-values for Figure 2c. The criterion to reject the null hypothesis after false discovery rate (FDR) adjustment (alpha = 0.01) was $p < 0.006$. (**b**) *P*-values for Figure 3a. The threshold for FDR adjustment was $p < 0.0058$.

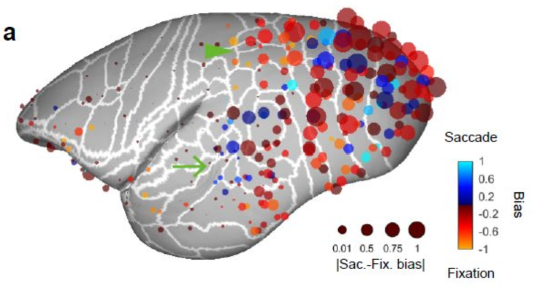

Supplementary Figure 2. Perisaccadic modulation time-locked with saccade or fixation onset

S-f bias indicates to what extent peak activation or suppression was time-locked to saccade or fixation onset (see the Methods section for details). (**a**) S-f bias for the activation peak. (**b**) S-f bias for the suppression peak. The s-f bias pattern was different between the dSTS and PPC/dorsal occipital regions (indicated by green arrows and wedges, respectively).

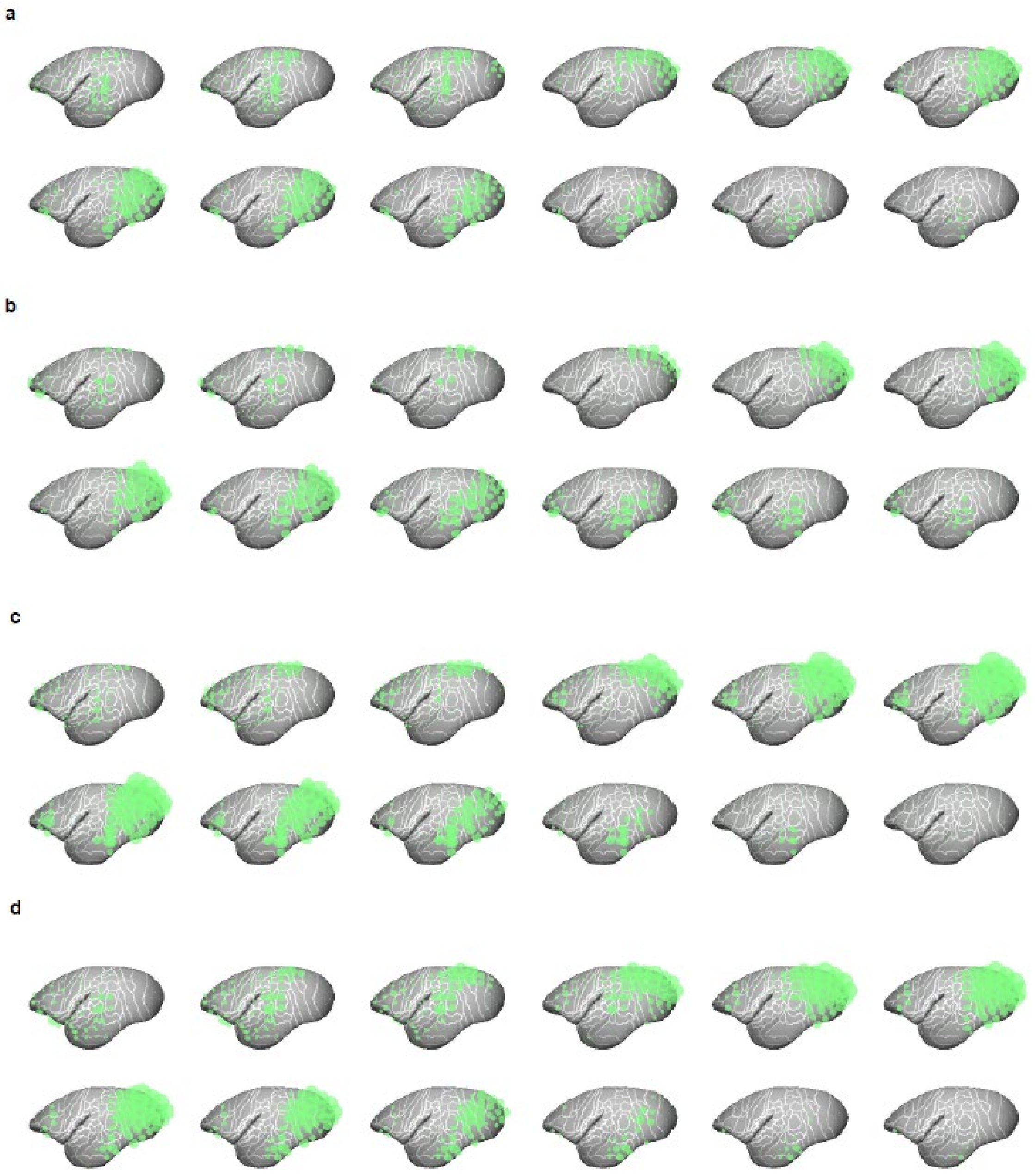

Supplementary Figure 3. Trajectory of the high-gamma signal on the cortical surface for individual data

(**a–d**) Magnitude of the high-gamma signal in each time bin around a saccade. These are individual data of Figure 6a. A consistent pattern of signal trajectory was observed for all subjects.

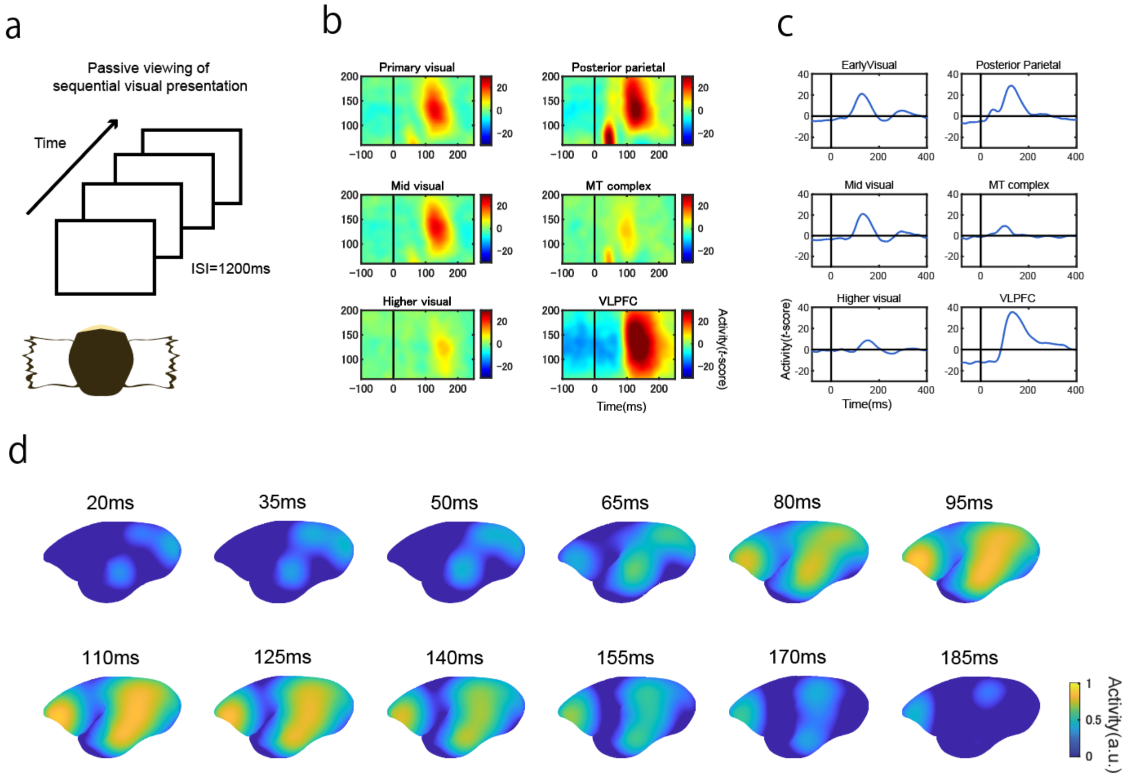

Supplementary Figure 4. Neural dynamics of passive visual perception

(**a**) Schematic illustration of the passive-viewing task. Static pictures of natural scenes were presented sequentially with an average stimulus interval of 1,200 ms. (**b**) Example spectrograms of the visually evoked responses. The ECoG signals were aligned by stimulus onset. (**c**) Time course of high-gamma (100–160 Hz) activity around stimulus onset for the same electrodes as in (b). (**d**) Spatio-temporal dynamics of the high-gamma signals after stimulus onset. In contrast to active vision (Fig. 4), the initial response emerged at the most posterior part of the occipital regions and dSTS and this expanded toward the temporal and parietal cortices, which correspond to the ventral and dorsal stream, respectively.

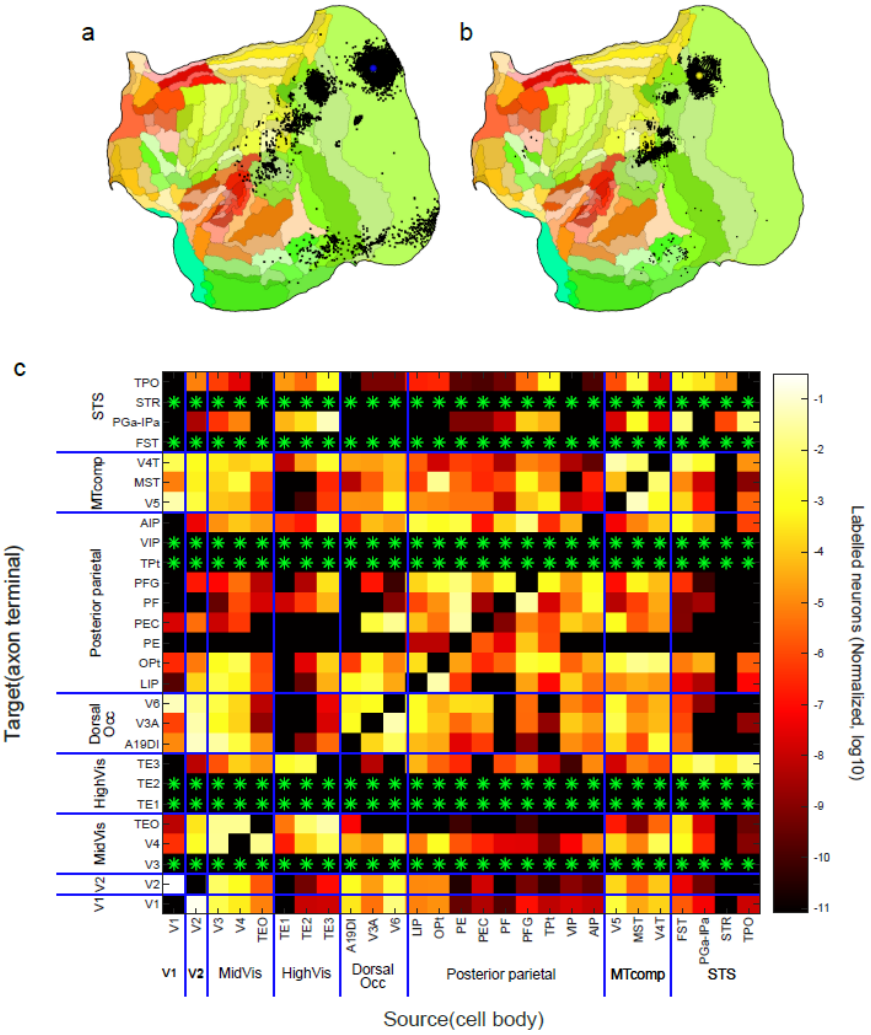

Supplementary Figure 5. Anatomical connectivity from an open database

Meta-analysis for retrograde tracer injections in the marmoset neocortex. Data derived from Majka et al[33]. (**a**) An example case of retrograde tracer injection in V1. The injection position is indicated by the blue dot. The labeled neurons are shown as small black dots on the flatmap of the marmoset cortex. The background colors show different cortical areas. V1 received axonal input from a variety of dorsal occipital and posterior parietal regions. (**b**) An example of retrograde tracer injection in V2. The injection position is indicated by the yellow dot. (**c**) The color code represents a fraction of extrinsically labeled neurons. Areas without any injection are marked by green asterisks. The connection matrix showed there was a significant number of axonal projections from the areas belonging to the dorsal stream (MT complex, dorsal occipital, and posterior parietal regions) toward the early visual areas such as V1 or V2.

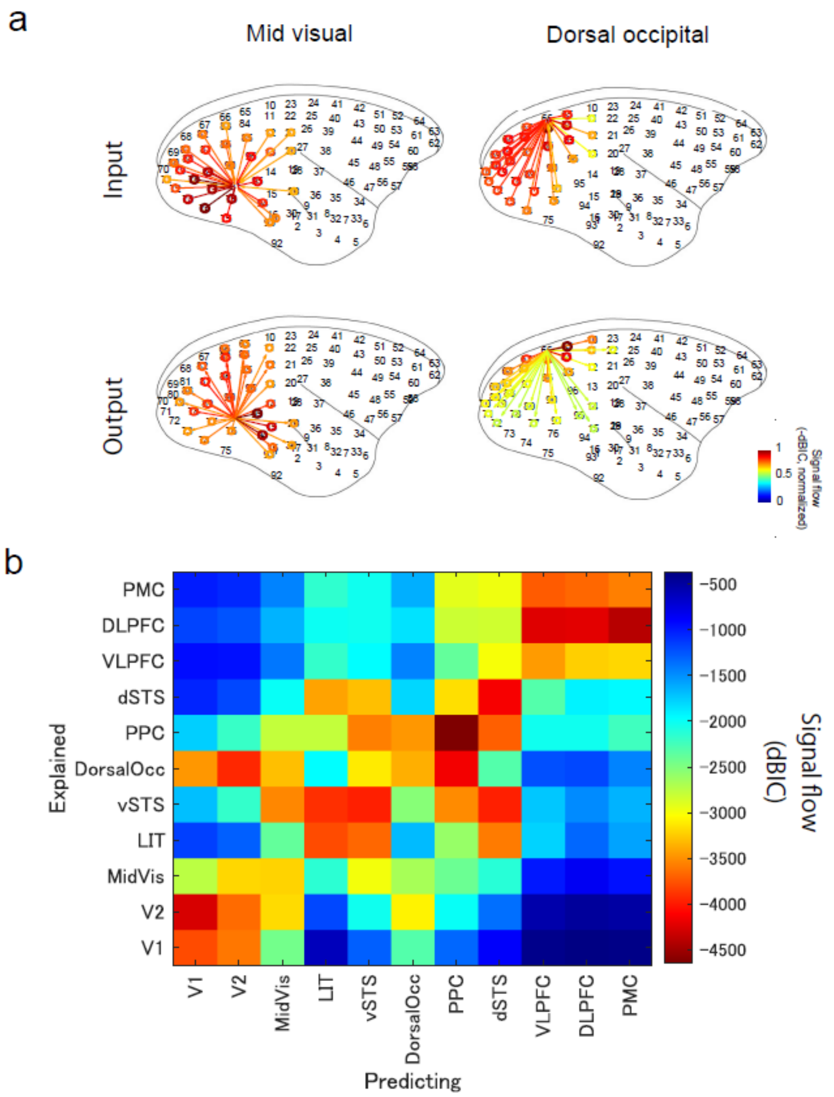

Supplementary Figure 6. Granger causality analysis

(**a**) Examples of Granger causality analysis of the high-gamma signals. In each brain map, Granger causality was color-coded for the pairs of electrodes with the top 30 combinations of high Granger causality. In the mid visual area, the input signal was largely derived from the early visual areas, and the output was more toward the anterior visual areas. In the electrodes of the dorsal occipital area, Granger causality was high from the early visual areas, but there was also a significant amount of Granger causality from the dorsal occipital to early visual areas. (**b**) Granger causality analysis across different cortical regions from 4 animals. The signal in the MT complex and PPC influenced the dorsal occipital regions, and the dorsal occipital regions also influenced the earlier visual areas.

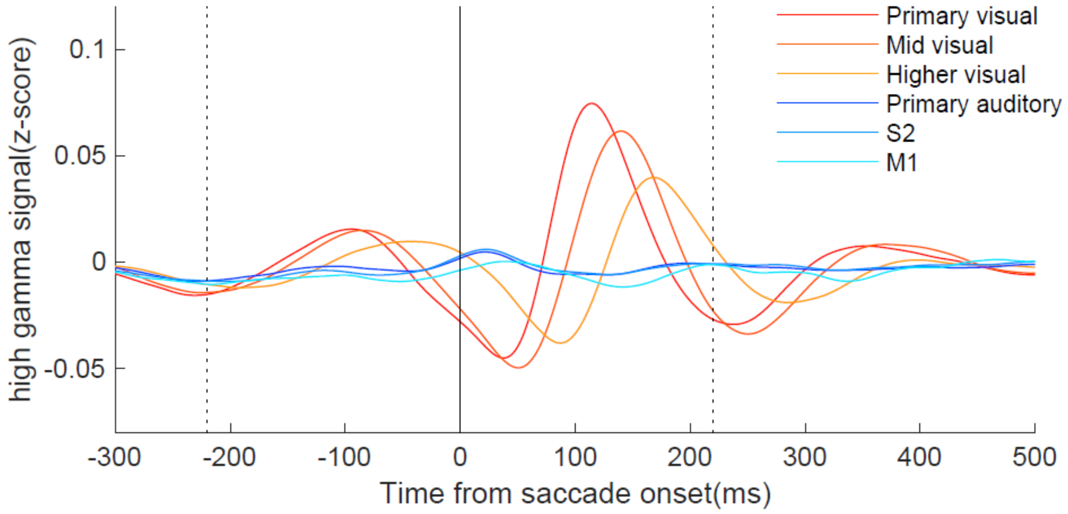

Supplementary Figure 7. Signal time course of visual and non-visuomotor areas

High-gamma signal time course of perisaccadic periods. Example electrodes with strong or weak (absent) saccadic modulation. Signal modulation of the non-visuomotor area was subtle compared to other visuo-motor areas, although it was not completely absent (see also Fig. 2c, Fig. 3a, Supplementary Fig. 1a, b).

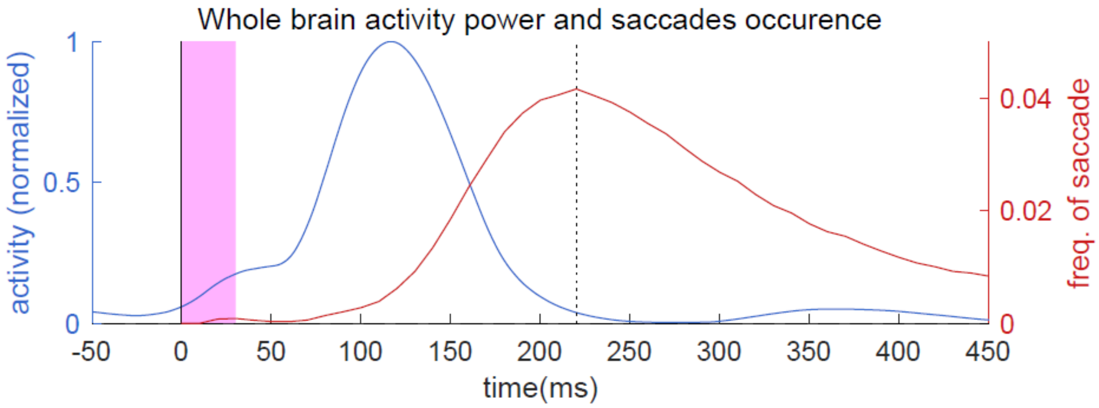

Supplementary Figure 8. Temporal dynamics of whole brain activity in relation to saccade interval (only visual responsive areas)

Temporal dynamics of whole brain activity, as in Figure 7b, except that this figure includes electrodes only from V1, V2, mid visual areas, lateral inferior temporal areas, vSTS, dSTS, dorsal occipital region, PPC, and VLPFC.

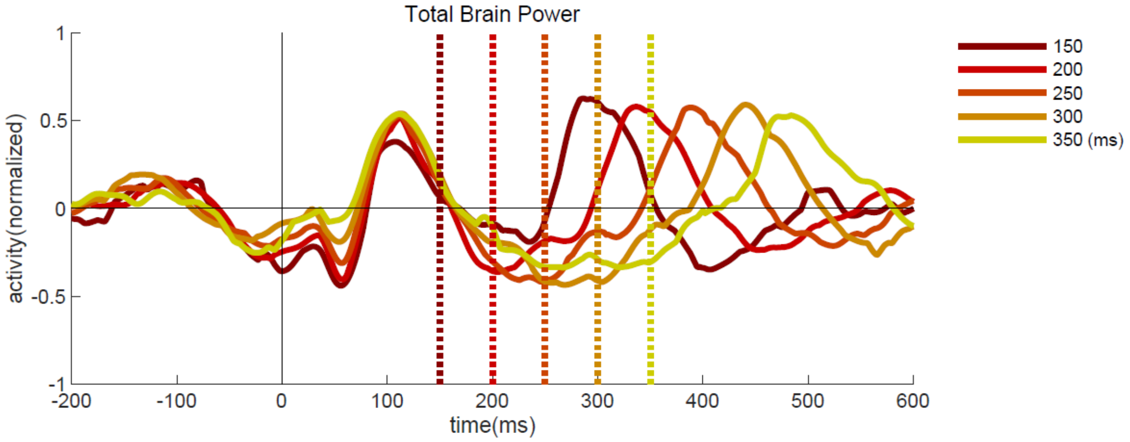

Supplementary Figure 9. Temporal dynamics of whole brain activity including positive and negative components

Same analysis as in Figure 8a, except that this figure includes the negative part of high-gamma activity. The signal was normalized by the difference between the minimum and maximum signals within the perisaccadic period to compare temporal dynamics across conditions. The 1st peak of activation derived from the saccade was used to align the data, and the 2nd peak was related to the saccade that terminated fixation. The temporal dynamics of the 1st peak were comparable across conditions in contrast to the change in its dynamics with the 2nd peak, suggesting that signal dynamics are not slowed or quickened regardless of subsequent fixation duration (in other words, a short fixation duration is not related to fast signal transmission). This observation was consist with our conclusion, i.e., the signal transmission pattern did not change according to subsequent fixation duration, and thus the activation pattern on the moment of saccade onset was drastically different according to prior fixation duration.

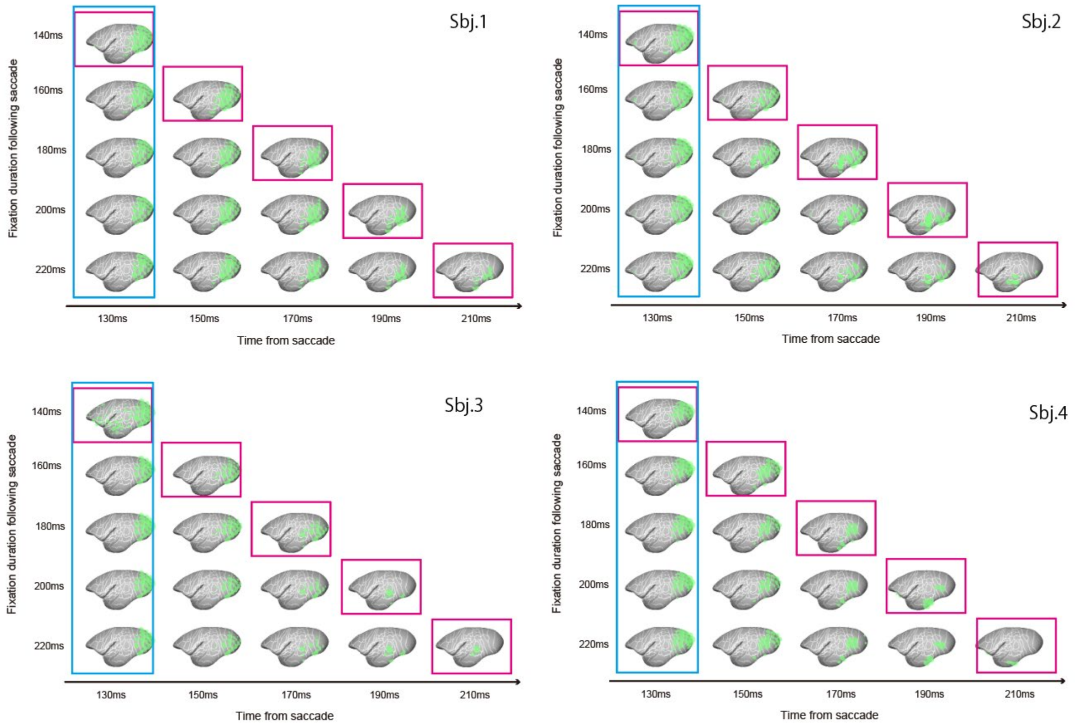

Supplementary Figure 10. Individual data for distinctive activity profiles at fixation termination for different fixation durations

Individual data on the activity patterns for saccades with different fixation durations as in Figure 8b. The overall trends were comparable across subjects, although there were some partial differences because of slight variation in the precise location of the electrodes.

Supplementary Table 1. Mapping cortical annotation to brain regions

When we reported latency in different brain regions (such as the higher visual areas or dSTS), we grouped the electrodes into brain regions based on three types of information: 1) the position of the electrode on the brain surface in MR and CT imaging; 2) cortical area parcellation inferred from atlas registration; and 3) the functional properties of electrodes (latency and activity strength). In this table, we listed the mapping across the cytoarchitectonic names in the atlas and our final grouping of the electrodes. We did not solely rely on atlas registration because the simple spatial deformation of MR imaging does not guarantee a precise inference of area annotation.

Region	Area	Region	Area	Region	Area
V1	V1	DorsalOcc	A19DI	dSTS	MST
V2	V2		V3A		MT
MidVis	V3		V6		dFST
	V4	PPC	LIP		STR
	TEO		Opt		TPO(STP)
LIT	TE1		PE	vSTS	vFST
	TE2		PEC		PG/IPa
	TE3		PF		V4t
			PFG		
			PG		
			TPt		
			VIP		
			AIP		
