# Supplementary material for: Cortical neural dynamics unveil the rhythm of natural visual behavior in marmosets
Source: Commun Biol. 2022 Feb 3;5:108. doi: 10.1038/s42003-022-03052-1 (PMC8814246; doi:10.1038/s42003-022-03052-1)
Supplement: Supplementary file 3 — Description of Additional Supplementary Files [file 42003_2022_3052_MOESM3_ESM.pdf]

Description of Additional Supplementary Files

File name: Supplementary Data 1
Description: Source data underlying Fig 2d, 3c, 4c, and 9d.
