# Supplementary material for: Cortical neural dynamics unveil the rhythm of natural visual behavior in marmosets
Source: Commun Biol. 2022 Feb 3;5:108. doi: 10.1038/s42003-022-03052-1 (PMC8814246; doi:10.1038/s42003-022-03052-1)
Supplement: Supplementary file 5 — Reporting Summary [file 42003_2022_3052_MOESM5_ESM.pdf]

nature research

Corresponding author(s): Takaaki Kaneko

Last updated by author(s): Dec 18, 2021

Reporting Summary

Nature Research wishes to improve the reproducibility of the work that we publish. This form provides structure for consistency and transparency in reporting. For further information on Nature Research policies, see our Editorial Policies and the Editorial Policy Checklist.

Statistics

For all statistical analyses, confirm that the following items are present in the figure legend, table legend, main text, or Methods section.

n/a	Confirmed	
[ ]	[x]	The exact sample size (*n*) for each experimental group/condition, given as a discrete number and unit of measurement
[ ]	[x]	A statement on whether measurements were taken from distinct samples or whether the same sample was measured repeatedly
[ ]	[x]	The statistical test(s) used AND whether they are one- or two-sided<br>*Only common tests should be described solely by name; describe more complex techniques in the Methods section.*
[x]	[ ]	A description of all covariates tested
[ ]	[x]	A description of any assumptions or corrections, such as tests of normality and adjustment for multiple comparisons
[ ]	[x]	A full description of the statistical parameters including central tendency (e.g. means) or other basic estimates (e.g. regression coefficient) AND variation (e.g. standard deviation) or associated estimates of uncertainty (e.g. confidence intervals)
[ ]	[x]	For null hypothesis testing, the test statistic (e.g. *F*, *t*, *r*) with confidence intervals, effect sizes, degrees of freedom and *P* value noted<br>*Give P values as exact values whenever suitable.*
[x]	[ ]	For Bayesian analysis, information on the choice of priors and Markov chain Monte Carlo settings
[x]	[ ]	For hierarchical and complex designs, identification of the appropriate level for tests and full reporting of outcomes
[x]	[ ]	Estimates of effect sizes (e.g. Cohen's *d*, Pearson's *r*), indicating how they were calculated

Our web collection on statistics for biologists contains articles on many of the points above.

Software and code

Policy information about availability of computer code

Data collection	We used iRecHS for eye-tracking(https://staff.aist.go.jp/k.matsuda/iRecHS2/index_e.html), and psychtoolbox-3 and Matlab(R2018a) for stimulus presentation and data acquisition.
Data analysis	We used custom codes written in Matlab(R2018a-R2021a) for data analysis. We also used AFNI(v17.2<), ANTs(v2<) and 3D-Slicer(v3<) for CT and MRI analysis to estimate the locations of the implanted electrodes.

For manuscripts utilizing custom algorithms or software that are central to the research but not yet described in published literature, software must be made available to editors and reviewers. We strongly encourage code deposition in a community repository (e.g. GitHub). See the Nature Research guidelines for submitting code & software for further information.

Data

Policy information about availability of data

All manuscripts must include a data availability statement. This statement should provide the following information, where applicable:

- Accession codes, unique identifiers, or web links for publicly available datasets
- A list of figures that have associated raw data
- A description of any restrictions on data availability

The data sets generated and/or analyzed in the current study are available from the corresponding author on reasonable request.

Field-specific reporting

Please select the one below that is the best fit for your research. If you are not sure, read the appropriate sections before making your selection.

- [x] Life sciences
- [ ] Behavioural & social sciences
- [ ] Ecological, evolutionary & environmental sciences

For a reference copy of the document with all sections, see nature.com/documents/nr-reporting-summary-flat.pdf

Life sciences study design

All studies must disclose on these points even when the disclosure is negative.

Sample size	The number of animals used in this study followed the standard in similar studies in non-human primates. We used a minimum number of animals to judge consistency across subjects. The number of sessions we recorded for ECoG signal is empirically determined based on the robustness and consistency of results in high-gamma signal.
Data exclusions	No data were excluded from the analyses.
Replication	All results we reported were consistent across the animals.
Randomization	Randomization was not relevant to this study. The experimental design in this study was a repeated measure so that the different conditions were tested in the same animals.
Blinding	Not applicable because no grouping was performed.

Reporting for specific materials, systems and methods

We require information from authors about some types of materials, experimental systems and methods used in many studies. Here, indicate whether each material, system or method listed is relevant to your study. If you are not sure if a list item applies to your research, read the appropriate section before selecting a response.

Materials & experimental systems

n/a	Involved in the study	
x		Antibodies
x		Eukaryotic cell lines
x		Palaeontology and archaeology
	x	Animals and other organisms
x		Human research participants
x		Clinical data
x		Dual use research of concern

Methods

n/a	Involved in the study	
x		ChIP-seq
x		Flow cytometry
x		MRI-based neuroimaging

Animals and other organisms

Policy information about studies involving animals; ARRIVE guidelines recommended for reporting animal research

Laboratory animals	Four male common marmosets(Callithrix jacchus), ranging from 1 year 10 months olds to 3 years 6 months olds, were used in this study.
Wild animals	No wild animals were used in this study.
Field-collected samples	No field collected samples were used in this study.
Ethics oversight	All procedures in the animal experiments were approved by the Wako Animal Experiment Committee (Animal Care and Use Committee), RIKEN, and all experiments followed the institutional ethical guidelines for the care and use of experimental animals.

Note that full information on the approval of the study protocol must also be provided in the manuscript.
